# Mortality of Surgical Operations in the Upper Lake States, Compared with That of Other Regions

**Published:** 1876-12

**Authors:** Edmund Andrews

**Affiliations:** Professor of Princples and Practice of Surgery in Chicago Medical College


### THE MORTALITY OF SURGICAL OPERATIONS IN THE UPPER LAKE STATES, COMPARED WITH THAT OF OTHER REGIONS.

By EDMUND ANDREWS, A M., M.D.,

PROFESSOR OF PRINCPLES AND PRACTICE OF SURGERY IN CHICAGO MEDICAL COLLEGE,

ASSISTED BY THOS. B. LACEY, M.D.,

ASSISTANT SURGEON IN THE NATIONAL SOLDIERS' HOME.

(Continued.)

### EXCISIONS OF THE WRIST JOINT.

I have occasionally performed this operation, but have preserved no records of the cases, nor obtained any from other Lake State surgeons.

Abroad the statistics are very meager, and of doubtful value.

I have gleaned from various works three hundred and five cases, with fifty-seven deaths, which is a mortality of nineteen per cent. Many of the cases were only partial, leaving the complex articulations of the remaining bones to suppurate and breed pyæmia in very unfavorable circumstances. It appears to me that complete resections would be far safer. The utter poverty of the literature on this subject compels us to lay it aside as not yet properly investigated.

Probably a complete resection would be advisable whenever it offers a fair opportunity to save a hand, whose innervation and circulation is in fair condition.

### RESECTIONS AT THE HIP JOINT.

Of these I have nineteen cases from the Lake States, with eight deaths, which is a mortality of forty-two per cent. They were all cases of caries of the joint. The literature of the profession furnishes us the following figures for other regions:

TRAUMATIC PRIMARY RESECTIONS OF THE HIP ABROAD.

| AUTHORITY. | CASES. | DIED. |
|---|---|---|
| Circular No. 2, S. G. O., p. 137, collected by Otis | 39 | 36 |

Mortality, 92 per cent.

TRAUMATIC INTERMEDIARY RESECTIONS OF THE HIP ABROAD.

| AUTHORITY. | CASES. | DIED. |
|---|---|---|
| Circular No. 2, S. G. O., p. 137, collected by Otis | 33 | 30 |

Mortality, 91 per cent.

PURELY SECONDARY RESECTIONS OF THE HIP ABROAD.

| AUTHORITY. | CASES. | DIED. |
|---|---|---|
| Circular No. 6, S. G. O., p. 137 | 13 | 11 |

Mortality, 85 per cent.

COMBINED INTERMEDIARY AND SECONDARY RESECTIONS OF THE HIP ABROAD.

| AUTHORITIES, | CASES. | DIED. |
|---|---|---|
| Warren, American Confed. Army | 1 | 0 |
| Chisholm, " " " | 2 | 1 |
| Billroth's Briefe | 2 | 2 |
| Deutsch. Zeit. f. Chir. Bd. I., S. 187 | 1 | 1 |
| Archiv. Klin. Chir., B. 13, S. 575 | 1 | 1 |
| Totals | 7 | 5 |

Mortality, 71 per cent.

Extensive statistics have been gathered on the Pathological Resections of the Hip Joint, by Hodges, Ashurst, R. Good, Heyfelder, Sayre, Fock, Leisrink and H. Lyster. These have been carefully collated by Ashurst, in the Pennsylvania Hospital Report, 1869, and in his Surgery, 1871. Gant, in his Surgery, published the same year, gives a collection of late cases from British hospitals, which appear to be mostly or entirely additional to those collated by Ashurst. Tho two collections taken together give a tolerable summary of what is known on the subject, and are here subjoined:

PATHOLOGICAL RESECTIONS OF THE HIP JOINT ABROAD.

| AUTHORITIES. | CASES. | DIED. |
|---|---|---|
| Ashurst's Surgery, p. 605; terminated cases | 327 | 163 |
| Gant's " p. 638; " " | 79 | 22 |
| Totals | 406 | 185 |

Mortality, 46 per cent.

The cases of Sayre and other American surgeons are included in Ashurst's collection, with many from Germany, France, etc. Gant's cases are all British, and show decidedly better results than Ashurst's figures, which are gathered from all nations. The French and German cases especially are very fatal.

GENERAL SUMMARY.

| | LAKE STATES. | | | ABROAD. | | |
|---|---|---|---|---|---|---|
| | CASE | DIED | PER CT. MORT | CASE | DIED | PER CT. MORT |
| Primary | ---- | ---- | ---- | 39 | 36 | 92 |
| Intermediary | ---- | ---- | ---- | 33 | 30 | 91 |
| Purely secondary | ---- | ---- | ---- | 13 | 11 | 85 |
| Combined intermediary and secondary | ---- | ---- | ---- | 7 | 5 | 71 |
| Pathological | 19 | 8 | 42 | 406 | 185 | 46 |

It appears, therefore, that in the Lake States we have no recorded experience of traumatic cases, but in pathological ones our results are somewhat better than the average given in surgical literature, and immensely better than in France, where Leisrink gives a mortality of eighty-six per cent. The seventy-nine late cases from British hospitals, however, give only twenty-eight per cent., which is better than the Lake States by eighteen per cent.

### OPINIONS OF AUTHORS.

Only a few years ago excisions of the hip joint for disease were generally condemned, but of late this opinion has been reversed.

Ashurst, (Penn. Hosp. Rept., 1869,) thinks it a serious operation, only to be undertaken when there is no reasonable prospect of recovery without it. He concludes:

1. That the sex of the patient is immaterial.
2. That the age is important, the success before puberty being much greater than after.
3. That total excisions are as successful as partial.
4. That in fatal cases only one-fourth of the deaths are due to the operation.
5. That in gunshot wounds (p. 211, Ashurst's Surgery,) fracturing the joint, excision, though very fatal, is the safest course.

Gant's Surgery, p. 632, claims that destruction of the articular cartilages of the hip without anchylosis always justifies the excision, (Mr. Hancock advocates the same practice,) but that the operation should not be performed for mere anchylosis. Mr. Gant says that disease of the acetabulum does not prohibit the operation, but that the effect of spontaneous dislocation is rather to be reckoned as opposed to excision. For gunshot fractures of the joint he seems to favor the excision, as being excessively fatal, yet about the only hope left the patient.

Prof. Sayre, of New York, says (*Orthopedic Surgery*, page 284), that in hip disease, with vitiated constitution and bones diseased beyond the operator's reach, the patient is probably hopeless; but when the disease is chiefly local, the constitution not undermined, the bones not affected beyond the possiblity of removal, and the circumstances of air, food, etc., favorable, the operation offers the best possible chances of recovery.

Holmes, of England, in direct contradiction to Sayre, thinks that the poor patients, located in bad air and under bad nursing, are the ones that ought to be operated on to relieve them, as quickly as possible, from the irritating diseased bone, while patients in good circumstances will have a better chance without operation.

Hamilton favors the operation in a suitable selection of carious cases, but doubts its applicability to gunshot wounds.

Gross, Erichsen, Druitt, Bryant, Holmes and Johnstone favor it in a proper selection of cases of hip disease.

Dr. J. S. Sherman, late Professor of Orthopedic Surgery and Diseases of the Joints in Chicago Medical College, says that in determining the question of excision for hip disease, "the general condition of the patient should be considered as much as the condition of the joint itself. Cases in which the general condition is bad, and which do not yield readily to treatment, should be operated on early, independent of the amount of caries; but where the general condition is good, and endurance can be expected, the operation should be postponed. The majority of such cases recover excellently without operation. Hence, it is justifiable to give them a chance and not exsect early, but if exhaustion occurs exsection is necessary."

Surgeon Otis, of the U. S. Army (Circular No. 2, S. G. O. P. 123), after the most elaborate view ever made of the subject of gunshot wounds of the hip, says:

"Primary excisions of the head and upper extremity of the femur should be performed in all uncomplicated cases of gunshot fractures of the head or neck. Intermediate excisions are indicated if the diagnosis is not made out till late, and also in gunshot fracture of the trochanter with consecutive arthritis. Secondary excisions are demanded by caries or secondary involvement of the joint from fractures or wounds in the immediate vicinity."

"Expectant treatment is to be condemned in all cases in which direct injury to the articulation can be clearly established."

Hamilton, as we have already mentioned, doubts the soundness of Otis' conclusion.

CONCLUSIONS.

In caries of the hip joint, below the age of puberty, excision is not a very dangerous operation; and, although the mortality is from twenty-five to forty per cent., yet only one-twelfth of the deaths are due to the operation. Almost always the patient is relieved, and if he dies it is in spite of the operation. Above the age of puberty the danger of the operation is much greater, and the majority of the patients die.

If this excision is contemplated, therefore, in a child approaching the age of puberty, it should be done as early as possible, and after that age should not be done at all if it can be avoided.

Formerly excision of the hip for caries was generally condemned, but some twenty years ago the old prohibitions were broken over, and numerous cases were operated on. As experience accumulated, however, it was found that though the operation rarely killed the patient, yet the wounds were very slow in healing, so that in many instances those that were not operated on recovered fully as early as those subjected to excision. This damped the ardor of the operators somewhat, and at the present time we seem to be forced to about the following conclusions:

1. Cases of morbus coxarius which do not suppurate, of course do not suggest any operation.

2. Suppurative cases, which do well under tonics, antiseptic injections, etc., should have a full and prolonged trial of expectant treatment, and only be operated on if their progress seems to be obstinately slow.

3. Cases below puberty, which gradually get worse, the fistulas refusing to heal, and the patient steadily losing ground, should be operated on reasonably early, without waiting for extreme exhaustion.

4. Cases above puberty may be operated on if no other hope exists, but not until the minor operation of opening the joint and using antiseptic injections and dressings has been thoroughly tried on Lister's plan.

5. Sex exerts little influence on the results.

6. Disease of the ilium, to a moderate extent, is no obstacle to excision.

7. When spontaneous luxation occurs it usually relieves the patient of a great irritation, and favors spontaneous recovery, but if otherwise it constitutes no objection to excision.

8. The great majority of cases of suppurating hip disease will recover without operation.

9. In uncomplicated gunshot fractures of the hip joint, the present state of science indicates that excision should be performed as soon as possible, yet nine-tenth of the patients are reported as dying; and if all the cases were known, which the chagrin of the surgeons caused them to suppress, the mortality would show still worse. It is greatly to be desired that more light should be had on this terrible subject. Surgeon Otis could scarcely find a single clear case of recovery from gunshot fracture of the hip without operation (Cir. No. 2, p. 121), yet the depth of the parts caused the diagnosis to be very doubtful in many cases; and we may well stagger at an operation whose deaths are an unknown quantity, ranging somewhere between ninety and one hundred per cent. of the whole.

Perhaps the antiseptic plan would give better results.

### RESECTIONS OF THE KNEE JOINT.

This operation has received little favor in the Lake States. I have records of only eight cases, of which three died. All the eight cases were pathological.

In other regions we have the following list. (The numbers being too few, and the records too imperfect, the traumatic cases cannot be classified into primary, intermediary and secondary. They are all for gunshot wounds and display a fearful mortality):

TRAUMATIC RESECTIONS OF THE KNEE JOINT, ABROAD.

| AUTHORITIES. | CASES. | DIED. |
|---|---|---|
| Circular No. 6, American Cases | 10 | 8 |
| " No. 6, Foreign " | 12 | 11 |
| Chisholm, Confederate Army, United States | 4 | 3 |
| Warren " " " " | 1 | 0 |
| Billroth, Arch. klin. Chir. B. X. und Briefe | 2 | 2 |
| Billroth's Briefe, p. 267 | 20 | 17 |
| Geissel, German-French War | 3 | 3 |
| Herrgolt, " " | 1 | 1 |
| Totals | 53 | 45 |

Mortality, 85 per cent.

PATHOLOGICAL RESECTIONS OF THE KNEE JOINT, ABROAD

| AUTHORITIES. | CASES. | DIED. |
|---|---|---|
| Boston City Hospt. Rept. | 6 | 1 |
| Cases Collected by Dr. R. Hodges, Boston | 208 | 60 |
| " " " M. L. Peniere, 1762 to 1869 | 431 | 131 |
| " " " Gant from British Hosps. Gants' Surgery p. 621 | 241 | 64 |
| Sundry German Operators | 15 | 9 |
| Totals | 901 | 265 |

Mortality, 29 per cent.

Some of these figures of different authors include, partly, the same cases as others, and their analysis does not enable me to separate them completely; but as the repetitions affect both columns alike, they do not materially change the ratio of mortality.

GENERAL SUMMARY OF RESECTIONS OF THE KNEE.

| | CASES. | DIED. | PER CT. MORT. |
|---|---|---|---|
| Lake States, Pathological | 8 | 3 | 37 |
| Traumatic, Abroad | 53 | 45 | 85 |
| Pathological, " | 901 | 265 | 29 |

It appears, therefore, that, contrary to our experience in amputations, pathological resections of the knee have been less successful here than abroad. However, the number of our cases is so small that this may be an accidental occurrence.

OPINIONS OF AUTHORS.

Hodges shows that excision of the knee has a higher mortality than amputation above it, and that out of 208 case 102 failed or died.

Bryant, of London, shows that of 431 cases of disease of the knee which were amputated, the mortality was only twenty-two per cent., while of 178 similar cases resected, thirty-nine per cent. died. After a careful reivew of the subject, he opposes the operation.

T. Holmes speaks discouragingly of the operation, but allows that it may, perhaps, be done in chronic disease of the knee, in patients below the middle age.

Gant favors excision cautiously in caries, provided the disease does not occupy too much of the bones. He endeavors to show that the mortality of excision of the knee is no greater than that of amputation of the thigh, and consequently preferable, because it saves the limb with no greater risk than is incurred in amputation. In this argument he commits the singular error of comparing the mortality of the excision with that of amputation of all parts of the thigh, high and low, alike. Now, the choice lies only between excision and amputation at the lower third of the thigh, where the mortality of amputation is much less than that of excision.

Pirogoff disapproves the operation in war surgery.

Hamilton discourages the operation, especially in gunshot wounds.

McLeod and Longmore oppose it in military practice.

Ashurst opposes it in gunshot cases, but allows it in disease. He says it is not very successful below five years of age, and very fatal in persons past the prime of life; but the safest period is from five years to puberty. Recent cases of disease should generally not be operated on; but only those which have either actually proceeded to caries and suppuration, or else show the characters of gelatinous arthritis (white swelling of old writers), in the doughy, semi-elastic swelling. In a suitable selection of such cases he recommends excision, but in those who are too old, or too young, or who have visceral complications, or too extensive disease of the bone to leave a useful limb after its excision, or who cannot afford the long time of recovery which excision requires, amputation is to be preferred. (*Med. Record*, Vol. 2, p. 443.)

Erichsen allows it doubtfully in extensive disease or faulty anchylosis.

Sir Geo. Ballingall favored it in civil, but opposed it in military practice.

Guthrie favored it in military practice, provided all the circumstances were favorable.

Gross approves it for disease, if the latter is not too extensive.

Butcher, Swain, Ferguson and P. H. Watson approve it strongly, and Druitt says it is one of the greatest triumphs of modern surgery.

CONCLUSIONS.

The above opinions are contradictory enough to satisfy the genius of discord. In such a confusion of precepts of the masters, we must appeal to the facts, which give us the following results:

| | PER CENT. |
|---|---|
| Mortality of Traumatic Amputations of the Lower Third of the Thigh, Abroad | 45 to 60 |
| Mortality of the Traumatic Excisions of the Knee Joint, Abroad | 85 |
| Mortality of Pathological Amputation of Lower Third of Thigh, Abroad | 20 |
| Mortality of Pathological Excision of the Knee, Abroad | 29 |

The figures of the Lake States, so far as they go, confirm the showing of a much greater mortality for the excision than for the amputation.

Eighty-five per cent. is a terrible death rate, and is sufficient to condemn the traumatic excision to final oblivion. The pathological excisions are only nine per cent. worse than the amputations, and this difference is not so great but it may be properly overruled in some cases where the importance of saving a natural limb is very great. The drawbacks, however, must be considered, and they are these:

1. Owing to the inflamed condition of the bones the time of healing is often ten or fifteen months.

2. There is always considerable doubt about such an anchylosis of the bones as will enable the patient to step on the limb.

3. A large per centage of cases fail so completely as to necessitate a subsequent amputation.

4. In children, if the operator removes the entire epiphysis of the femur, the growth of the limb in length is, in a great measure, arrested.

A candid consideration of all these facts renders it impossible for the conscientious surgeon to recommend excision of the knee joint in more than a very few unusual cases.

The eight pathological excisions of the knee in the Lake States are too small a number to yield any special conclusions, yet the three deaths in the eight cases are a dismal recommendation when compared with our twenty-two pathological amputations of the lower third of the thigh, without a single death.

### RESECTIONS OF THE ANKLE JOINT.

Of these the Lake States furnish us nine cases with one death, which is eleven per cent.

Abroad the literature is scanty, so that the subdivision of traumatic cases is impossible. Taken together they are as follows:

TRAUMATIC RESECTIONS OF THE ANKLE, ABROAD.

| AUTHORITIES. | CASES. | DIED. |
|---|---|---|
| Deutsch. Zeit. B. V., S. 26 | 1 | 0 |
| " " B. I., S. 187 | 2 | 2 |
| " " B. II., S. 106 | 5 | 3 |
| Stromeyer, Battle of Langensalza | 1 | 0 |
| Heyfelder, Resectionen, p. 162 | 67 | 6 |
| Circular No. 6, S. G. O. | 18 | 6 |
| Jaeger's Tables | 29 | 1 |
| Sir Astley Cooper | 9 | 0 |
| Josse | 6 | 0 |
| Taylor | 5 | 0 |
| Gants' Collection | 4 | 1 |
| Totals | 147 | 19 |

Mortality, 13 per cent.

PATHOLOGICAL RESECTIONS OF THE ANKLE, ABROAD.

| AUTHORITIES. | CASES. | DIED. |
|---|---|---|
| Hancock's Collection of British Cases | 34 | 2 |
| Heyfelder's Collection, deducting British Cases, Resectionen, p. 162 | 25 | 3 |
| Archiv. klin. Chir. B. 8 und 10 | 10 | 3 |
| Deutsch Zeit. B. II., S. 380, Lücke | 3 | 1 |
| Totals | 72 | 9 |

Mortality, 12 per cent.

OPINIONS OF AUTHORS.

Hueter thinks resection of the ankle is indicated in suppurative inflammation of the joint.

Langenbeck had a "run of bad luck" with it, all his eight cases performed for caries being failures; however, he recommends it for many cases of gunshot wounds. He and the German surgeons generally favor the sub-periosteal method.

Mayer opposes partial resections, but A. Rose and Frank Hamilton favor them when circumstances demand.

Ashurst, p. 612, brings statistics to show that excision of the external malleolus has the same mortality as that of the entire ankle, viz.: twenty per cent.

Pirogoff believes resection of the ankle to be safer than amputation in compound fractures, and that the risk of conservative treatment is intermediate between the two.

Kade disapproves the operation in most cases.

Gant, p. 644, favors the operation for disease, provided the affected portions of the bones do not extend too far from the joint. In gunshot wounds of the ankle, he generally prefers excision to amputation, p. 289.

Ashurst, pp. 211 and 612, favors both traumatic and pathological excisions.

Holmes, in the first edition of his System of Surgery, opposed the operation, but in the second edition retracts his opinion, and cautiously favors excision.

Gross advocates it where the caries is not too extensive.

Erichsen and Druitt both oppose it.

CONCLUSIONS.

As compared with amputation of the leg in the lower third, excision of the ankle is much the safest.

The following figures show the difference:

| | Mort. of Exc. of Ankle, Abroad. | Mort. of Amp. of lower 3d of Leg, Abroad. |
|---|---|---|
| Traumatic | 13 per cent. | 33 per cent. |
| Pathological | 12 " | 16 " |

In the Lake States the operation has proved a little safer than abroad, viz.:

| | | |
|---|---|---|
| Average Mortality of all Excisions of Ankle, Abroad | 12½ | per ct. |
| " " " " " Lake States | 11 | " |

As between excision and amputation there is only one conclusion possible. Excision is far the safest, and also preserves a useful foot for walking. It is therefore to be preferred to amputation whenever the choice is possible; that is, when the foot is not mortified, nor the parts above and below the joint too much diseased or disorganized to allow their serving a future useful purpose. It is here to be remembered that Lister's antiseptic treatment has revolutionized some of our precepts. I have, by this method, healed an ankle proved by the probe to be completely carious, though I am sure that one could not always succeed in that way. It would be well in many cases to give it a trial before proceeding to excision. In the same antiseptic way, many compound dislocations and fractures of the joint can be readily cured without operation.

On the whole the following statement probably gives pretty nearly the truth:

CASES FOR AMPUTATION.

1. Death of the foot from any cause.

2. Cancer or incurable disease of the foot, rendering its presence pernicious.

3. Caries and necrosis, so destroying the parts above and below the joint as to render it impossible to remove the disease and have the end of the tibia rest on the foot, or to preserve periosteum enough to reproduce the bones.

4. Compound fractures effecting similar destruction of parts.

CASES FOR EXCISION.

1. All ordinary cases of caries, which resist antiseptic treatment.
2. Certain cases of talipes, and displacement from old accidents, which resist orthopedic treatment.
3. Compound fractures and dislocations which have resisted antiseptic measures, and have not destroyed the circulation in the foot.
4. Dislocations of the astragalus, and compound dislocation of the tibia forward which will not remain in position after being reduced.

CASES FOR ANTISEPTIC TREATMENT.

1. Caries in its earlier suppurative period.
2. Simple suppuration of the joint.
3. Compound fractures and dislocations which will stay reduced, which have not destroyed the circulation in the foot, nor hopelessly disorganized too much of the adjacent parts.

OPERATIONS UPON THE LARNYX AND TRACHEA.

Of these I have obtained trustworthy records of thirty-five cases, some of which are of decided interest:

### TABLE XIII.

### Operations on the Larynx and Trachea.

| No. Andrew's Sur.Rec | OPERATOR. | Age. | REASON FOR OPERATION. | COMPLICATIONS. | OPERATION. | Cond. at opr | Time to operation. | Result. | Time to death or recovery | Practice. |
|---|---|---|---|---|---|---|---|---|---|---|
| | Dr. H.A. Johnson | 2 | Memb. croup | None | Trache.abv.thyroid gld. | Bad | 3 days | Died | 1 hour | Private |
| | " " " | 6 | Diphtheria | None | Laryngotomy | " | 4 or 5 days. | " | 1 " | " |
| | " " " | 4 | Memb. croup | None | " | " | | " | | Hospital. |
| 116 | " E. Andrews | 1 | Diphtheria | None | " | " | | " | 6 weeks | Private |
| 138 | " H. A. Johnson | 1 | " | None | " | " | | " | 24 hours | " |
| 674 | " E. Andrews | 12 | Oedema glotidis | None | " | Good | | Recover'd | | " |
| 7593 | " " " | 2 | Foreign body in trachea | None | Trache. below thyroid. | " | 1 or 2 days. | " | | " |
| 7592 | " " " | | " " | None | " " | Bad | some hours | Died | 1 hour | " |
| 7594 | " " " | 3 | " " | None | " in haste | " | " | " | 10 min | " |
| 7595 | " H. A. Johnson | 7 | Diphtheria | None | " above thyroid | Med. | | " | 4 days | " |
| 7598 | " E. Andrews | 7 | " | None | Laryngotomy | Bad | | " | 3 " | " |
| 7599 | " " " | 3 | " | None | " | " | | " | 3 " | " |
| 8003 | " " " | 40 | Stricture of glottis with asphyxia. | Phthisis | Trache. above thyroid. | " | | Successful | | Hospital. |
| | " Herrick | 4 | Oedema " | None | " | | | Recover'd | | |
| | " D. Brainard | 1 | Abscess at root of tongue | None | " | | | " | | |
| | " " " | 2 | Foreign body in trachea 3 months | | " | | | " | | |
| | " H. A. Johnson | 3 | Diphtheria | None | " above thyroid. | Bad | 4 days | " | | Private |
| | " " " | 3 | Memb. croup | None | " " | " | 1 " | Died | 24 hours | " |
| | " H. Wardner | 1 | " | None | " | " | 8 " | " | 5 " | " |
| | " " " | 1 | " | None | " | " | 6 " | Recover'd | 120 " | " |
| | " " " | 3 | " | None | " | " | 7 " | Died | 12 " | " |
| | " H. A. Johnson | 2 | " | None | " above thyroid. | " | 48 hours | " | 10 " | " |
| | " E. Powell | 30 | Oedema of larynx | Oedema of lungs | Laryngo-tracheotomy | " | 16 days | " | 26 " | Hospital. |
| | " J. W. Freer | | Foreign body in larynx | None | Trache. below thyroid. | " | 2 weeks | Recover'd | 14 days | " |
| | " J. Andrews | 2 | Foreign body in trachea | | " | Good | | " | | Private |
| | " Gunn | 3 | Diphtheria | None | " | Bad | 10 days | " | 28 days | " |
| | " R. G. Bogue | | " | None | " | | | " | | " |
| | " H. A. Johnson | 7 | Epithelioma in larynx | None | High trache. Tumor removed twice, 2d sucesfl | Med. | 1 year | " | | " |
| | " " " | 3 | Diphtheria | None | Trache. above thyroid. | Bad | 4 days | Died | 1 hour | " |
| | " " " | 10 | " | None | " " " | Bad | 8 " | Died | 10 hours | " |
| | " " " | 60 | Cancer in larynx | None | " " " | Med. | 5 months | Lived | 3 mos. | " |
| | " " " | 5 | Diphtheria | Art. mortis | " " " | Bad | 7 days | Died | few hrs. | " |
| | " " " | 6 | Memb. croup | None | " " " | Fair | 5 " | " | 23 hours | " |
| | " " " | 4 | " | None | " " " | " | 7 " | " | 22 " | " |
| | " " " | 40 | Swelling of laryngeal muc. memb. | Ulc. larynx | " " " | " | Not stated. | Lived | | " |

RECAPITULATION.

| | CASES. | DIED. | PER CENT. MORTALITY. |
|---|---|---|---|
| Total for all causes | 35 | 20 | 57 |
| For memb. croup or diphtheria | 21 | 17 | 81 |
| For foreign bodies in trachea | 6 | 2 | 33 |
| For Oedema glottidis | 4 | 1 | 25 |
| For other causes | 4 | 0 | 0 |

The diphtheritic portion of the cases, arranged by age, are as follows:

| | CASES. | DIED. |
|---|---|---|
| Under 1 year | 3 | 2 |
| 1 to 2 years | 3 | 3 |
| 2 to 3 " | 4 | 3 |
| 3 to 4 " | 3 | 2 |
| 4 to 5 " | 1 | 1 |
| 5 to 6 " | 2 | 2 |
| 6 to 7 " | 2 | 2 |
| 7 to 8 " | 1 | 1 |
| 8 to 9 " | 0 | 0 |
| 9 to 10 " | 1 | 1 |

The case of epithelioma of the larynx was a remarkable one. The growth obstructed respiration, so that asphyxia was imminent. Prof. H. A. Johnson then performed tracheotomy. The tumor continued to grow until it encroached downward upon the lower part of the larynx; the patient still wearing the tracheotomy tube. Prof. Johnson then divided the thyroid cartilage in the middle line, and removed the growth, which had its seat just below the vocal chords. The wound healed nicely, the patient still wearing the tracheotomy tube. After a considerable period the growth returned, and Prof. Johnson repeated the thyrotomy, removed the tumor more thoroughly, and cauterized the seat of it with nitric acid. The recovery was good, and the surpising part is that after two operations of thyrotomy, the patient has a good and constantly improving use of the vocal chords in speaking. The last operation was many months ago. The tumor has not returned. Its character as an epithelioma was thoroughly demonstrated by the microscope.

(To be contined.)

